# The impact of early aging on visual perception of space and time

**DOI:** 10.3389/fnhum.2022.988644

**Published:** 2022-11-16

**Authors:** Sara Incao, Carlo Mazzola, Alessandra Sciutti

**Affiliations:** ^1^Cognitive Architecture for Collaborative Technologies Unit, Italian Institute of Technology (IIT), Genoa, Italy; ^2^Dipartimento di Informatica, Bioingegneria, Robotica e Ingegneria dei Sistemi (DIBRIS), University of Genoa, Genoa, Italy; ^3^Department of Robotics, Brain and Cognitive Science, Italian Institute of Technology (IIT), Genoa, Italy

**Keywords:** context dependency, visual perception, temporal perception, spatial perception, early aging, regression to the mean, Bayesian models, central tendency

## Abstract

Visual perception of space and time has been shown to rely on context dependency, an inferential process by which the average magnitude of a series of stimuli previously experienced acts as a prior during perception. This article aims to investigate the presence and evolution of this phenomenon in early aging. Two groups of participants belonging to two different age ranges (Young Adults: average age 28.8 years old; Older Adults: average age 62.8 years old) participated in the study performing a discrimination and a reproduction task, both in a spatial and temporal conditions. In particular, they were asked to evaluate lengths in the spatial domain and interval durations in the temporal one. Early aging resulted to be associated to a general decline of the perceptual acuity, which is particularly evident in the temporal condition. The context dependency phenomenon was preserved also during aging, maintaining similar levels as those exhibited by the younger group in both space and time perception. However, the older group showed a greater variability in context dependency among participants, perhaps due to different strategies used to face a higher uncertainty in the perceptual process.

## Introduction

The perception of space and time is very relevant for everyday life: consider the number of spatial and temporal estimations made when driving a car or when crossing a road. To make inferences about the world, humans base their predictions on past experience. Our knowledge of phenomena previously observed is the key to face the uncertainty derived by sensory experience. Hence, the perceptual process can be seen as an integration between the information coming from the senses and prior experience organized in internal models that act as priors. An example of such integration is represented by context dependency, also known as central tendency (Helmholtz, 1866; Hollingworth, 1910; Jazayeri and Shadlen, 2010; Karaminis et al., 2016; Roach et al., 2017). This phenomenon describes the way the predictive model is formed while perceiving a series of stimuli: the perception of each stimulus is influenced by the previous ones, so that the overall perception of the whole series of stimuli gravitates toward a mean magnitude. For instance, when we are shown several long segments and then we are asked to judge the length of another, shorter, one, we will perceive this one as longer than its actual size. This is because the perception is based on the prior, that can correspond to the average of the stimuli perceived before.

Context dependency has been observed in different perceptual domains and across different senses. These include auditory perception of time intervals (Jazayeri and Shadlen, 2010; Cicchini et al., 2012; Karaminis et al., 2016; Roach et al., 2017), visual perception of lengths (Sciutti et al., 2014; Mazzola et al., 2020), of points in space (Bejjanki et al., 2016), of categories (Huttenlocher et al., 2000) and of objects (Kersten and Yuille, 2003), and visual speed perception (Weiss et al., 2002; Stocker and Simoncelli, 2006). It has been shown that the integration process between sensory inputs and past experience can be modeled in Bayesian terms (Cicchini et al., 2012; Sciutti et al., 2014; Karaminis et al., 2016). According to this model, the more uncertain the sensory input is, the more relative weight is given to prior knowledge. Therefore, a great uncertainty in the sensory input should lead to a higher reliance on the prior, i.e., on stimulus history, and hence to a greater regression to the mean. Although some studies have investigated context dependency during development (Sciutti et al., 2014; Karaminis et al., 2016; Hallez et al., 2019), to our knowledge the phenomenon has been explored in aging in the temporal domain only by Gu et al. (2016). The present study aims at bridging this gap by assessing whether and how context dependency in the visual perception of space and time is influenced by the early phases of aging. Gu et al. (2016) focused their research on temporal memory as a function of temporal context dependency. To this aim, they designed the reproduction tasks with stimuli ranging from 7 s to 14 s, a time interval which is beyond the threshold of the psychological present (Fraisse, 1984). In our study, instead, we wanted to estimate how context dependency affected participants’ perception by using shorter stimuli ranging from 1.27 s to 1.8 s as in Karaminis et al. (2016) with an additional discrimination task as a perceptual acuity assessment. In particular, we focused on changes occurring in people still active in society. For this reason, we selected participants with a mean age of about 60 years, who were still active in their work or in other activities that require a high perceptual and cognitive load. Regarding the visuo-spatial perception, and particularly the ability to visually discriminate lengths, differences between younger and older adults are reportedly not significant (Norman et al., 2014; Billino and Drewing, 2018). Also, Latham and Barrett (1998) found no age-related effect in a spatial discrimination task. In their experiment, participants had to decide whether the separation of the stimuli (distance between two white luminance patches) presented in the first interval was longer or smaller than the one presented in the second interval. However, as Faubert (2002) pointed out, visuo-spatial perceptual processes are affected by aging in case of increasing cognitive demand required by more complex tasks, such as delayed matching tasks or the processing of more than a single attribute per stimulus. This result is consistent with Lemay et al. (2004), which found that the difference between older and younger participants is visible only in the condition that required the stimulus to be remembered before executing the movement to reproduce the length of the target. They hypothesize that the information about target location was no longer available in the iconic memory of older participants having the stimulus been presented 1.5 s before the reproduction phase. The ability to visually discriminate distances seems to be preserved with age growing unless the tasks involve cognitive demands, in particular working memory, or specific mechanisms of integration, a circumstance that could be traced to the processing of multiple attributes of a single stimulus.

As for what concerns visuo-temporal perception, one of the most evident differences with increasing age is the higher variability in the elderly’s answers (Wittmann and Lehnhoff, 2005; Turgeon et al., 2016; Lamotte and Droit-Volet, 2017). For instance, Turgeon et al. (2016) suggest that greater is the age, more variable are the tapping rates in different tapping tasks. In regard to time sensitivity, it has been demonstrated that perceptual acuity gradually declines with increasing age (Lamotte and Droit-Volet, 2017; Scurry et al., 2019; Mioni et al., 2021). Specifically, Lamotte and Droit-Volet (2017) found a decline in time sensitivity in the older group (76–81 years) with a bisection task. Mioni et al. (2021) assessed a worsening in perceptual acuity with a time discrimination task employing comparison intervals of 0.5 or 1.5 s. They found that when the standard stimulus was 1.5 s (similar to the design of our study: 1.535 s), the main differences already occurred from the age of 45. Gu et al. (2016) and Mioni et al. (2020) underlined a decline in time reproduction accuracy. In Mioni et al. (2020), besides an increased variability, older subjects showed a general tendency to underestimate their temporal judgments when asked to reproduce a time interval but to overestimate them in time production task. By contrast, Gu et al. (2016) found an effect of context dependency leading the aged group to a higher accuracy bias of the reproduced duration with respect to the stimuli. In addition, the role of cognitive functions is believed to account for the age-related changes in temporal perception. For example, mechanisms of attention and working memory that should decline with advanced age, play a fundamental role in evaluating the changes in perception of time for older adults (age range: 60–80 years old; Baudouin et al., 2006; Bartholomew et al., 2015; Brown et al., 2015).

The objective of this study was therefore to investigate how visual perception of space and time evolves in early aging in terms of perceptual acuity and use of priors. From previous literature and the Bayesian model of Context Dependency, the expectation is to find a stronger regression to the mean for the older adults in visual time perception to compensate an increased sensory uncertainty. Conversely, similar degrees of context dependency are expected for space perception between the two different age groups.

## Materials and Methods

The aim of the study was to evaluate whether the mechanism of context dependency in visual perception of space and time undergoes a change throughout life. To address this question, we asked participants from two different age groups to perform six tasks. Three tasks were designed to investigate the perception of space and three to assess the perception of time.

### Participants

Forty-seven participants in total were recruited for this study. Twenty-five participants were classified as “*Young adults*” (YA), 12 males, 13 females (*M* = 28.8 years old, SD = 4.6). Twenty-two participants were classified as “*Old adults*” (OA) nine males, 13 females (*M* = 62.8 years old, SD = 4.1). The age range in the OA group was selected to include participants who could potentially exhibit age-related decline in visual perception, while maintaining good motor and cognitive abilities. In the *OA* group, one participant was excluded for the impossibility to complete the task, leaving a sample of 21 participants (eight male and 12 female). The study was approved by the regional ethical committee and all participants provided written informed consent before participating. All participants had normal or corrected-to-normal vision.

### Task design

The experiment was divided in two conditions: space and time. Both conditions comprised three tasks. The order of conditions and tasks was randomized between participants. The experiment was performed in rooms lighted only with a lamp with a 11.5-Watt and 92 lm/W bulb placed near the screen, but pointing at the wall in front of the participant. The low-light condition was designed to avoid reflections on the screen. In both conditions, the participant sat on a chair with no wheels at a distance of 60 cm from the screen that was placed on a table (height 75 cm). The experiment was programmed and run with MATLAB 2019a and Psychtoolbox on a Windows 10 pc (Dell Inspiron 14 5000 2-1). In the space condition tasks, the stimuli were shown on a touchscreen ELO 2002L 20” monitor (resolution of 1,920 × 1,080 px for an active area of 436.9 mm × 240.7 mm, at a frequency of 60 Hz and Response Time of 0.02 s), whereas in the time condition tasks stimuli were shown directly on the Dell laptop screen (resolution of 1,920 × 1,080 px for an active area of 309.35 mm × 173.99, at a frequency of 60 Hz).

#### Space condition tasks

##### Pointing execution error—control task

Since the experiment included a reproduction task, we wanted to verify whether there were any significant differences between the two age groups in terms of motor abilities. For this reason, we designed a task where the stimuli were always visible and the participants were instructed to reach and touch them with their finger. For 50 trials, participants saw a red dot equal to the ones of the other spatial tasks, appearing in a random position on the screen. The number of possible positions was five in total: upper left corner, upper right corner, lower left corner, lower right corner (all of them 2 cm distant from the frame of the screen) and center of the screen. The participant had to touch the screen at the center of the red dot with as much precision as possible. The dot remained visible until the participant had completed the touch. The accuracy of the touch was measured as the distance between the center of the red dot and the touch of the participant.

##### Space discrimination task

**Figure 1:**
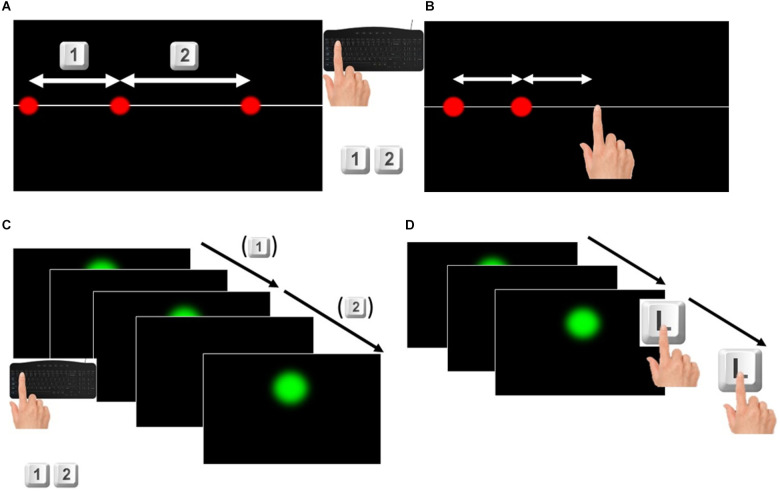
**(A)** Space discrimination task. For each trial participants were shown three red dots appearing simultaneously without any time interval between them. They were asked to judge which one was the longer and to press respectively the keys 1 or 2 on the keyboard if the longer was the one on the left or on the right. **(B)** Space reproduction task. For each trial participants were shown two red dots appearing consecutively without any time interval between them. They were asked to touch the touchscreen at the right of the second dot, to reproduce the distance between the two dots, by taking the second dot as reference. **(C)** Time discrimination task. For each trial participants were shown three green dots appearing at a certain time interval one from the other. They were asked to judge which one of the two time intervals was longer pressing respectively the keys 1 or 2 if the longer interval was the first (elapsed between the first and the second dot appeared) or the second (elapsed between the second and the third dot appeared). **(D)** Time reproduction task. For each trial participants were shown two green dots appearing at a certain time interval one from the other. They were asked to touch twice the letter L on the keyboard to reproduce the time interval between the two dots, pressing the start and the end of the time interval.

This task was designed to assess the perceptual acuity of participants. Three red dots of 1 cm diameter were shown simultaneously for 0.4 s on a white straight line crossing the screen at its central height. When the stimulus disappeared, participants had to judge whether the longest segment was the first—delimited by the first dot and the second one—or the second—delimited by the second dot and the third—pressing respectively the “1” or “2” button on the keyboard (see Figure 1A). One of the lengths was always 10 cm (standard) while the other (comparison) was showed according to the QUEST adaptive procedure (Watson and Pelli, 1983): starting value: 12.0 ± 3.6 cm (SD).

##### Space reproduction task

The set-up of this task was the same as the previous one, but the red dots appearing were two, determining a segment of a certain length. The participant was asked to reproduce the same length between the first and the second dot, touching the screen in a third point so that the distance between the first and the second dot was the same as the distance between the second dot and the touch of the participant (see Figure 1B). The first red dot appeared at a distance from the left border of the screen, ranging from 0.2 to 1.7 cm randomly selected. Six sets of 11 different lengths randomly shown were presented to each participant for 66 trials. The lengths were ranging from 6 to 14 cm, increasing each 0.8 cm as in (Sciutti et al., 2014; Mazzola et al., 2020). No clues about the correctness of the answers were given to participants.

#### Time condition tasks

##### Rhythm synchronization task—control task

This task was performed in order to measure participants’ ability to follow a constant rhythm, following a visual signal on the screen. The task consisted in following the rhythm marked by an intermittent green dot appearing on the screen for 50 trials. The green dot had a diameter of 2.2 cm and was placed 7.5 cm above the center of the screen. The participants were instructed to only look at the intermittent green dots for the first four appearances and internalize the rhythm. Then, they had to start pressing a keyboard key in order to synchronize the keypress with the appearance of the green dot.

##### Time discrimination task

This task was designed in a similar way to the space condition. Three green dots (2.2 cm diameter) appeared on the screen for 0.2 s defining two different time intervals: the first interval between the first and the second dot, the second interval between the second and the third dot (see Figure 1C). One interval—the standard—was constant (1.535 s) while the comparison interval was defined according to a QUEST adaptive procedure (Watson and Pelli, 1983): starting value: 1.7 ± 0.52 s (SD). The first dot was presented on the screen at a randomly varying time interval from the start of the trial, ranging from 1 s to 1.8 s. The number of trials performed by participants was not less than 50 but could vary according to the QUEST adaptive procedure.

##### Time reproduction task

The set-up of this task was similar to the previous one, but the dots (2.2 cm diameter, appearing on the screen for 0.2 s) were only two, therefore showing a single time interval. The participant had to reproduce this time interval pressing twice the letter L on the keyboard, so as to simulate the start and end (see Figure 1D). In a similar way to the space reproduction task, a set of 11 different time intervals, ranging from 1.270 s to 1.8 s as in (Karaminis et al., 2016) was shown six times and randomized within each set of stimuli, for a total of 66 trials. The first dot appeared on the screen after a varying delay from the start of the trial ranging from 1 to 2 s.

### Data analysis

#### Control tasks

For the pointing execution error task, we computed the distance between the stimulus shown on the screen (s) and the point touched by participants (r).


$$\text{Pointing err. =}\sqrt{{\left(x_{r}-x_{s}\right)}^{2}+{\left(y_{r}-y_{s}\right)}^{2}}$$

For the rhythm synchronization task, we measured participants’ ability to reproduce a rhythm by calculating the standard deviation of the time intervals indicated by the keypress.


$$\text{Rhythm Variability = }\sqrt{\frac{\sum_{i=1}^{N}{\left(x_{i}^{'}-\bar{x}\right)}^{2}}{N}}$$

where $x_{i}^{'}$ is the time interval between two consecutive keypresses. Since this measure was only relative to the temporal condition, a Wilcoxon Mann-Whitney test was used to compare the results of the two populations (YA, OA).

#### Discrimination tasks

**Figure 2:**
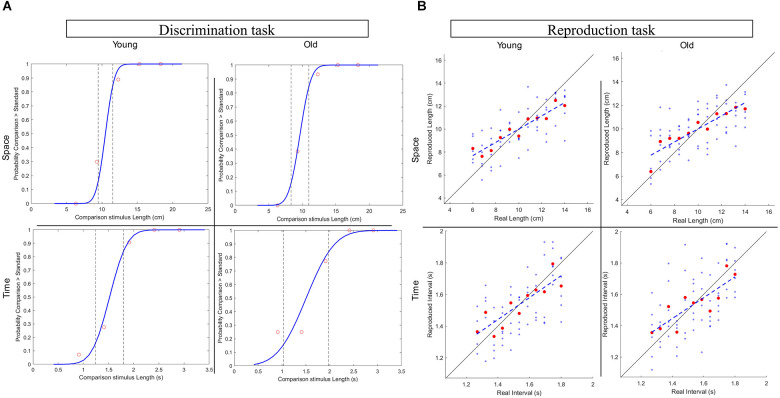
**(A)** Representative subjects’ plots for the Discrimination task in the spatial and temporal conditions from the two age groups. **(B)** Representative subjects’ plots for the Reproduction task in the spatial and temporal conditions from the two age groups.

For space and time condition, the differential threshold of each participant, that is the minimal difference between two lengths or time intervals that participants could reliably discriminate, was calculated as the standard deviation of the psychometric function (cumulative gaussian) fitted on the data of the discrimination tasks (see Figure 2A). Then, perceptual acuity was expressed as Weber Fraction (WF) measured as the ratio between the threshold and the standard stimulus (Cicchini et al., 2012; Karaminis et al., 2016).

#### Reproduction tasks

In the reproduction tasks, we evaluated both the average and the absolute perceptual bias of participants. Specifically, the offset was calculated by subtracting the average stimulus of all the trials ($\bar{S}$) from the average response ($\bar{R}$) to indicate participants tendency to overestimate or underestimate stimuli.


$$\text{Offset = }\bar{R}-\bar{S}$$

Well-established approaches were followed to estimate the degree of central tendency in spatial and temporal perception (Cicchini et al., 2012; Sciutti et al., 2014). As a direct measure of central tendency, we computed the regression index for each of the reproduction tasks (*Space Reproduction, Time Reproduction*) as the difference in slope between the identity line (ideal correct responses on stimuli) and the best linear fit of the responses given by the participant (participant’s responses plotted against the correspondent stimuli), see Figure 2B. Moreover, we also calculated the overall perceptual error (RMSE) as the root mean square between the accuracy error (BiasCD) and the precision error (CV). For each: (i) of the 11 stimuli of the Reproduction tasks, the degree of accuracy (BiasCD) results from the difference between the responses average (*R*_*Mi*_) and the corresponding stimulus (*S*_*i*_), normalized for the average stimulus ($\bar{S}$). The precision error is computed as the coefficient of variation (CV), namely the ratio between the standard deviation of the responses of a stimulus and the average stimulus.


$$BiasCD_{i}=\frac{\left(R_{Mi}-S_{i}\right)}{\bar{S}}$$

$${\text{CV}}_{i}=\frac{\sqrt{\frac{\sum {\left(R_{i}^{'}-\bar{R_{i}^{'}}\right)}^{2}}{N}}}{\bar{S}}$$

$${\text{RMSE}}_{i}=\sqrt{BiasCD_{i}^{2}+{\text{CV}}_{i}^{2}}$$

Following previous studies on context dependency (Cicchini et al., 2012; Sciutti et al., 2014; Karaminis et al., 2016; Mazzola et al., 2022), all these errors are calculated for each participant after subtracting from the participants’ responses (R) their Offset. In this way, the Offset is considered a perceptual offset caused by the individual tendency to perceive stimuli as greater or lower, independently from the stimulus history.


$${\text{R}}^{'}=\text{R}-\text{Offset}$$

Seven Linear Mixed Effect Models have been used to compare the effects of the conditions (Space and Time) and of the population (YA and OA) on the values of the Weber Fraction, the Offset, the Regression Index, and the three perceptual errors connected to context dependency (BiasCD, CV, and RMSE). These statistical analyses were conducted using R software (R i386 4.0.3) and specific libraries for Linear Mixed Effect Models (Kuznetsova et al., 2017; Lüdecke, 2021). The above mentioned parameters (Weber Fraction, Offset, Regression Index, Bias CD, CV, RMSE) were inserted as dependent variable, the condition (space and time), the age (*YA, OA*), and their interaction as predictors, and the subjects as random effect.

## Results

In this experiment we wanted to observe whether the visual perception of space and time and the central tendency mechanism supporting it are influenced by early aging in two representative age groups: YA and OA. In particular, we assessed potential variations in perceptual acuity and whether they had an impact on the use of prior knowledge in perception and on the participants’ perceptual bias.

### Control tasks

As regards the spatial control task, no significant difference was found in the pointing error when comparing the YA group (*Mdn* = 0.195 cm) with the OA group (*Mdn* = 0.243 cm) in a Mann-Whitney test: *U*_(25,21)_ = 262.50, *z* = −1.61, *p* = 0.107. This means that the basic motor abilities required to execute the spatial tasks were not significantly different with increasing age.

The rhythm synchronization task provided a measure of participants’ ability to synchronize to a given rhythm. To investigate this, it was measured the variability of time intervals reproduced by participant while attempting to follow the predefined rhythm. A high variability indicates participant’s difficulty in keeping up the pace with the rhythmic stimulus that was shown. Due to a technical problem, the data of two participants of the YA group for the rhythm synchronization task were not saved. A Mann-Whitney test on the remaining sample did not reveal any difference between the YA group (*Mdn* = 0.064 s) and the OA group (*Mdn* = 0.082 s): *U*_(23,21)_ = 295, *z* = −1.245, *p* = 0.213.

### Perceptual acuity

**Figure 3:**
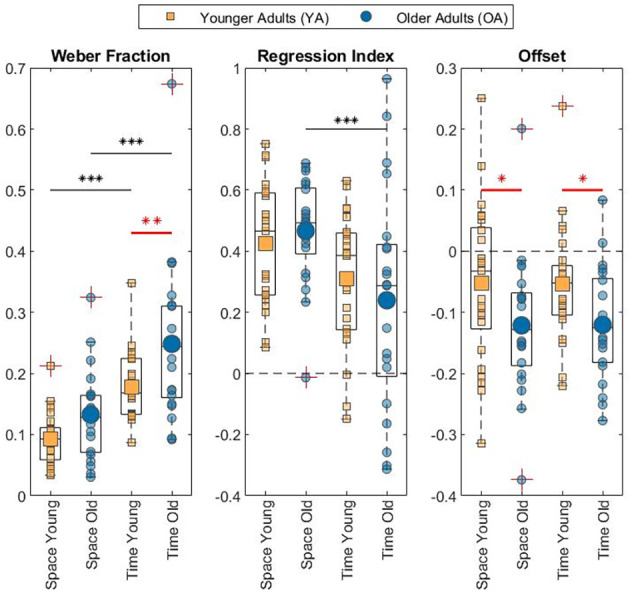
Box plot with individual data of the measures related to perceptual acuity (Weber Fraction), regression to the mean (Regression Index), and Offset. The asterisks mark statistical significance. Black ones denote difference between conditions. Red ones denote difference between age groups. *p < 0.05; **p < 0.01; ***p < 0.001.

The results of the discrimination tasks revealed that the Weber Fraction of the two populations of our study differs significantly only in the time condition (see Figure 3 for data visualization, Table 1 for means and Table 2 for statistics). Indeed, the Linear Mixed Effect Model on the WF showed that the temporal perception threshold is significantly higher in the OA group (YA-OA: *B* = −0.071, *t* = −2.998, *p* = 0.004). The same test does not reach significance for the spatial condition (YA-OA: *B* = −0.04, *t* = −1.673, *p* = 0.098), even though the trend is the same (see Table 1). In addition, from the same Linear Mixed Effect Model, in both YA and OA groups, the comparison between the Weber Fractions of space and time condition (Space-Time) resulted significantly different (YA: *B* = −0.084, *t* = −4.649, *p* < 0.001; OA: *B* = −0.116, *t* = −5.84, *p* < 0.001). This shows that, independently of their age, participants found the temporal discrimination task more difficult than the spatial one. No effect of interaction between condition and age was found. Moreover, it was not found any correlation between conditions for either groups.

**Table 1:** Means and Standard Deviations of the perceptual measures explored in the study for the two age groups.

**Age**	**Weber fraction**		**Offset**		**Regression index**		**Bias context dependency**		**Coefficient of Variation (CV)**		**Root Mean Squared Error (RMSE)**	
	Space	Time	Space	Time	Space	Time	Space	Time	Space	Time	Space	Time
YA	*M* = 0.093	*M* = 0.177	*M* = −0.053	*M* = −0.054	*M* = 0.426	*M* = 0.320	*M* = 0.119	*M* = 0.067	*M* = 0.114	*M* = 0.141	*M* = 0.165	*M* = 0.157
	SD = 0.043	SD = 0.056	SD = 0.128	SD = 0.092	SD = 0.195	SD = 0.193	SD = 0.045	SD = 0.014	SD = 0.022	SD = 0.040	SD = 0.029	SD = 0.039
OA	*M* = 0.133	*M* = 0.248	*M* = 0.121	*M* = −0.120	*M* = 0.467	*M* = 0.294	*M* = 0.131	*M* = 0.073	*M* = 0.121	*M* = 0.137	*M* = 0.174	*M* = 0.156
	SD = 0.074	SD = 0.129	SD = 0.114	SD = 0.092	SD = 0.168	SD = 0.294	SD = 0.036	SD = 0.024	SD = 0.037	SD = 0.05	SD = 0.043	SD = 0.050

**Table 2:** Results of the linear Mixed Effect Models for the seven perceptual measures of the discrimination and the reproduction tasks.

**Results of the 7 Linear Mixed Effect Models**													
			**condition: space-*time*/age: YA-*OA***						**condition: time-*space*/age: OA-*YA***				
			**Estimate**	**St. Err.**	**df**	**T**	***p***		**Estimate**	**St. Err.**	**df**	**T**	***p***
Weber Fraction (WF)	Intercept		0.248	0.017	78.109	14.245	<0.001		0.093	0.016	78.109	5.828	<0.001
	Condition		-0.116	0.020	44.000	-5.844	**<0.001**		0.084	0.018	44.000	4.649	**<0.001**
	Age		-0.071	0.024	78.109	-2.998	**0.004**		0.040	0.024	78.109	1.673	0.098
	Age*Condition		0.031	0.027	44.000	1.167	<0.249		0.031	0.027	44.000	1.167	0.249
Offset	Intercept		-0.120	0.024	88.000	-5.109	<0.001		-0.053	0.022	88.000	-2.443	0.017
	Condition		-0.001	0.033	88.000	-0.026	0.979		-0.001	0.031	88.000	-0.029	0.977
	Age		0.067	0.032	88.000	2.088	**0.040**		-0.069	0.032	88.000	-2.143	**0.035**
	Age*Condition		-0.002	0.045	88.000	0.039	0.969		0.002	0.045	88.000	0.039	0.969
Regression Index (RI)	Intercept		0.240	0.053	88.000	4.509	<0.001		0.426	0.049	88.000	8.751	0.000
	Condition		0.227	0.075	88.000	3.023	**0.003**		-0.116	0.069	88.000	-1.688	0.095
	Age		0.070	0.072	88.000	0.976	0.332		0.041	0.072	88.000	0.563	0.575
	Age*Condition		-0.111	0.102	88.000	-1.088	0.280		-0.111	0.102	88.000	-1.088	0.280
Bias Context Dependency (BiasCD)	Intercept		0.073	0.007	87.746	10.421	<0.001		0.119	0.006	87.746	18.579	<0.001
	Condition		0.058	0.010	44.000	6.074	**<0.001**		-0.052	0.009	44.000	-5.905	**<0.001**
	Age		-0.006	0.009	87.746	-0.618	0.538		0.012	0.009	87.746	1.289	0.201
	Age*Condition		-0.006	0.013	44.000	-0.488	0.628		-0.006	0.013	44.000	-0.488	0.628
Coefficient of Variation (CV)	Intercept		0.137	0.008	72.285	16.731	<0.001		0.114	0.008	72.285	15.192	<0.001
	Condition		-0.017	0.008	44.000	-1.977	0.054		0.027	0.008	44.000	3.422	<**0.001**
	Age		0.004	0.011	72.285	0.320	0.750		0.006	0.011	72.285	0.564	0.575
	Age*Condition		-0.010	0.012	44.000	-0.855	0.397		-0.010	0.012	44.000	-0.855	0.397
Root Mean Squared Error (RMSE)	Intercept		0.156	0.009	77.621	17.661	<0.001		0.165	0.008	77.621	20.377	<0.001
	Condition		0.018	0.010	44.000	1.805	0.078		-0.008	0.009	44.000	-0.929	0.358
	Age		0.000	0.012	77.621	0.041	0.967		0.009	0.012	77.621	0.751	0.455
	Age*Condition		-0.010	0.014	44.000	-0.703	0.486		-0.010	0.014	44.000	-0.703	0.486

The left column gives the statistics if taking as reference the temporal condition and the OA group, whereas the right column if taking as reference the spatial condition and the YA group. Significant *p* values for age, condition and age*condition predictors are in bold.

### Context dependency

For both conditions, the two age groups exhibited a regression to the mean during the reproduction task (see Table 1 for Mean and SD). Indeed, all Regression Indexes resulted significantly different from 0 in one-sample *t*-tests: YA Space: *t*_(24)_ = 10.95, Cohen’s *d* = 2.189, *p* < 0.001; OA Space: *t*_(20)_ = 12.57, Cohen’s *d* = 2.742, *p* < 0.001; YA Time: *t*_(24)_ = 7.26, Cohen’s *d* = 1.452, *p* < 0.001; OA Time: *t*_(20)_ = 3.02, Cohen’s *d* = 0.66, *p* = 0.007.

Focusing on the difference between age groups, no significant RI variations were found (see Figure 3 for data visualization, Table 1 for means and Table 2 for statistics) neither in the spatial perception, nor in the temporal one. Nevertheless, in the OA group, only for the temporal domain, RI increased significantly with growing age: *F*_(1,19)_ = 5.27, *R*^2^ = 0.22, *p* = 0.033.

As regards the difference between conditions, the Linear Mixed Effect Model of Regression Index showed that in the OA population the regression index was significantly lower in the visual perception of time than space (Space-Time: *B* = 0.227, *t* = 3.023, *p* = 0.003). Even though in the YA group no difference was found across conditions, the trend was the same (Space-Time: *B* = 0.116, *t* = 1.688, *p* < 0.095).

**Figure 4:**
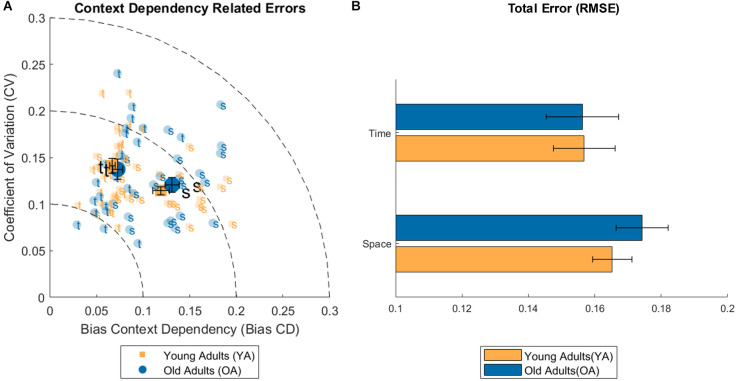
**(A)** Plot of the individual data for Bias and coefficient of variation (CV) related to the context dependency phenomenon (error bars represent the Standard Error of the mean). **(B)** Bar plot of the root mean squared error (RMSE) for the spatial and temporal condition and both age groups (error bars represent the Standard Error of the mean).

Concerning the three measures of perceptual errors connected to context dependency (BiasCD, CV, RMSE), see Figure 4, a significant difference between conditions has been found for the BiasCD (see Table 1 for means and Table 2 for statistics; Space-Time, YA: *B* = 0.052, *t* = 5.905, *p* < 0.001; OA: *B* = 0.058, *t* = 6.074, *p* < 0.001), revealing that both the age groups were more accurate in the temporal dimension. A significant variation, but in the opposite direction has been found for the OA group, also in the CV, demonstrating a loss in precision in the temporal domain (Space-Time, YA: *B* = −0.027, *t* = −3.422, *p* = 0.001). The same trend is visible also for the YA group (OA: *B* = −0.017, *t* = −1.977, *p* = 0.054). No difference between conditions has been found for the RMSE. Only the OA group exhibited a decreasing trend for the error in the temporal perception (Space-Time, OA: *B* = 0.018, *t* = 1.805 *p* = 0.078), although demonstrating a lower perceptual acuity (higher WF) in the discrimination task. No significant difference has been found between age groups for the three perceptual errors: neither in the spatial dimension, nor in the temporal one, despite the variation of perceptual acuity in time between younger and older adults. Moreover, no effect has been found for the interaction between age and condition, neither for the regression index, nor for the three perceptual errors related to context dependency. No correlation was found between conditions for either groups.

### Perceptual reproduction offset

Independently from the phenomenon of context dependency, also the measures of the average Offset was calculated from the results of the reproduction tasks. All the Offset means resulted significantly different from 0 in both conditions and for both age groups in one-tailed *t*-tests and revealed that, on average, participants underestimated both the temporal and the spatial stimuli. Specifically, the Offset in space in the YA group: *t*_(24)_ = −2.06, *p* = 0.05, Cohen’s *D* = −0.41, the Offset in time in the YA group: *t*_(24)_ = −2.91, *p* = 0.008, Cohen’s *D* = −0.58, the Offset in space in the OA group: *t*_(20)_ = −4.86, *p* < 0.001, Cohen’s *D* = −1.06, and the Offset in time in the OA group: *t*_(20)_ = −5.99, *p* < 0.001, Cohen’s *D* = −1.31 (means and the SDs as reported in Table 1).

Linear Mixed Effect models assessed the variation of Offset for condition and age group. For the Offset, the statistical analysis showed a significant effect of Age in both conditions, which revealed the OA group perceived stimuli as shorter with respect to the YA group (see Figure 3 for data visualization, Table 1 for means and Table 2 for statistics; YA-OA, Space: *B* = 0.069, *t* = 2.143, *p* = 0.035; Time: *B* = 0.067, *t* = 2.088, *p* = 0.040). No effect has been found between conditions, nor for the interaction between Age and Condition. Moreover, it was not found any correlation between conditions for either groups.

## Discussion

According to the aim of our research, we explored the effect of the early phases of aging on visual perception of space and time. The results indicate a general decline in perceptual acuity in the OA. Going deeper into the analysis of the two conditions, while for the spatial domain it is noticeable only with a slight decreasing trend, for the temporal domain, a significant difference between the two age groups emerged, which is also consistent with Mioni et al. (2021). In the selected temporal and spatial ranges, temporal discrimination resulted to be more difficult than spatial discrimination for both YA and OA groups, with Weber Fraction almost two times greater in the temporal than in the spatial condition. All participants had either normal or corrected-to-normal vision through the use of glasses. Such correction granted that all participants had the same potential capability to see the spatial stimuli presented in the task.

In this study, we also explored the phenomenon of context dependency in both domains of space and time. Such phenomenon intervenes when the information coming from the senses is uncertain and unprecise. Given the uncertainty of information, relying on our prior experience helps to reduce the variability of what is perceived. In accordance with previous research on the topic (Cicchini et al., 2012; Sciutti et al., 2014; Karaminis et al., 2016; Mazzola et al., 2022), we observed the mechanism of regression to the mean in both conditions, revealing a context dependency effect. In addition to the previous findings, this study found the phenomenon to be present also in an older population.

The close relation between visual acuity and use of priors has been found and modeled in a Bayesian fashion by previous studies (Cicchini et al., 2012; Sciutti et al., 2014; Karaminis et al., 2016; Mazzola et al., 2022). According to this model the decline of visual acuity we observed in the temporal domain with increasing age, would predict an increase in prior reliance. By contrast, the results of the time reproduction task did not reveal a higher regression index for the OA group compared to the younger group.

The transitional phase of the aging of the older population in this study may account for this result. Though the comparison between the two groups did not follow our predictions, age was shown to be a predictor for the RI within the OA group, such that in the temporal domain the older the subject, the higher the RI. From this perspective, one may speculate that if the decay of perceptual acuity is already visible from the age of 60 [in Mioni et al. (2021) already from 45], this same age is not sufficient to determine a consistent increase in the RI.

A different explanation is related to the mode of stimuli presentation. As Droit-Volet et al. (2008) demonstrated, differences in sensitivity of time, number, and length are only due to the sequential or nonsequential mode of presentation. In their experiment, when number and length were presented sequentially, as time is for its own nature (i.e., extended, with a duration), differences were leveled. Authors linked these findings with the higher attentional and cognitive resources required in the sequential presentation. Hence, these findings, combined with suggestions from previous literature (Faubert, 2002; Bartholomew et al., 2015; Lamotte and Droit-Volet, 2017), which indicate a worsening of elderly people’s perceptual performances when a higher attentional and cognitive control is required, are consistent with our results from the discrimination task. In the spatial (nonsequential) condition, we did not find any significant difference between the two age groups, which instead was found in the temporal (sequential) condition.

Following Droit-Volet et al. (2008), since worse attentional and cognitive control causes a decline in perceptual acuity, we can hypothesize this receptive difficulty be present also in the reproduction task of our study, somehow affecting the prior formation. Here, the sequential mode of presentation may have influenced the phenomenon of context dependency at the level of stimuli reception. Considering the design of the reproduction tasks, in the spatial domain there is no temporal interval between the presentation of the first and second dot forming the stimulus. By contrast, in the time reproduction task, the onset and offset of the stimulus are spaced out by a certain temporal interval and therefore higher attentional and cognitive load is required. As a result, a great variability is visible among older participants in the time reproduction task and the phenomenon of context dependency, as indicated by the RI, is weaker than what expected. The case is different for the spatial condition. Here, when the stimuli presentation is nonsequential, context dependency seems not to be affected by growing age. In general, context dependency was shown to be present with age growing but its mechanism may be impacted in case of a higher attentional and cognitive demand.

The Offset is another measure to analyze the effect of aging in the reproduction of time. It represents the mean of perceptual bias, providing also the information about its direction. Both in the spatial and the temporal conditions, the data showed a general tendency to underestimate the stimuli amplitude with a negative Offset that becomes broader with increasing age. Regarding the spatial domain, it was not possible to connect the underestimation strategy found in the OA group with other spatial perceptual measures of this study. By contrast, the decrease of the Offset (underestimation) in the OA group is consistent with their decline in perceptual acuity. A feasible explanation for the temporal underestimation in the reproduction task might be offered by the hypothesis of the internal clock model (Grondin, 2010). This theory considers the presence of a main mechanism responsible for temporal estimation and explains the representation of time in terms of pulses emitted by an internal clock. Previous literature supports the idea of a slower internal clock in the elderly (Turgeon et al., 2016; Lamotte and Droit-Volet, 2017). A slower clock would be due to fewer pulses emitted and therefore counted, a phenomenon that in reproduction tasks results in an underestimation of durations (Perbal et al., 2002). In the context of this hypothesis, the rhythm synchronization task may provide an interesting insight. During this task, the visual feedback of the stimulus was always present on the screen providing a reference. Conversely, during the reproduction task participants could only rely on their internal clock to reproduce the time interval. Interestingly, no difference between the two age groups was found in the rhythm synchronization task. Hence, the visual perception of time seems to be affected by the increasing age only when no visual feedback is provided, i.e., whether participants can only rely on their internal clock. As explained by Marinho et al. (2018), the variation of the internal clock is strictly connected with the dopaminergic system. Consequently, the stronger underestimation of OA group in the temporal reproduction task may be motivated by the decline in dopaminergic modulation that is showed to be present in older age (Li et al., 2010).

The motivation at the basis of this study was to understand whether and how visual perception of space and time changes with the increase of age. We wanted to focus on the age range in which sensory perception already undergoes a significant degradation, but the life of a person is still very active and similarly demanding, in terms of spatio-temporal abilities, as a younger age. Around 60 years of age, indeed, most people are still working or performing a rich range of activities. For the spatial domain, our data show that the overall performance of the older group was quite similar to the younger adults in the context of visual perception of space, both in terms of spatial acuity and regression to the mean. Only a general tendency to underestimate spatial amplitudes in the reproduction task differentiated significantly young and older adults. Conversely, temporal visual acuity resulted significantly reduced in the older adult group, together with a similar general underestimation of temporal intervals in the reproduction task. These findings indicate that already early during aging, visual perception of time undergoes significant changes. Focusing on the phenomenon of context dependency, in general, it appears clear that, with increasing age, in the temporal domain, the phenomenon of context dependency occurs differently than what would be expected from a direct application of the Bayesian modeling. In particular, in face of a significant reduction of their perceptual acuity, participants in the early aging group did not increase their tendency to rely on their prior.

Although our study is not definitive with respect to the causes underlying such variation, two possible directions emerge. First, it might be the case that with an older population, the expectations of the Bayesian model will be confirmed. Hence, a study across three different ages, adding an older population might be of help. Second, the sequential mode of presentation, the role of attentional/cognitive effort and the relation between space and time need further investigation. Modifying the mode of presentation might reveal whether a sequential spatial task and a temporal task present the same perceptual difficulties. Regarding the discrimination, this could result in a difference between age groups in the perceptual acuity. Whereas in regard to the reproduction, it may lead to a deviation from the Bayesian predictions of context dependency. Furthermore, adding sequentiality in a spatial task could also help in determining whether both age groups are affected by similar cognitive challenges at the level of context dependency. In this perspective, a test for cognitive performance, which was not present in our experiment, could shed light on the impact of cognitive and attentive control regardless of participants’ age. Leveling the cognitive difficulties among conditions might therefore be a possibility to deepen mechanisms connecting reception of stimuli, cognitive demand and the phenomenon of context dependency. Eventually, further research in this direction would be also crucial to understand whether in the elderly the deviation from Bayesian predictions is due to the higher cognitive and attentional demand, to different processes underlying spatial and temporal perception, or to other factors connected with aging.

## Data Availability Statement

The data supporting the conclusions of this article will be made available by the authors, without undue reservation.

## Ethics Statement

The studies involving human participants were reviewed and approved by Comitato Etico Regione Liguria. The participants provided their written informed consent to participate in this study.

## Author Contributions

SI and AS conceived and planned the experiments. SI and CM carried out the experiments and analyzed the data. All the authors contributed to the interpretation of the results. SI wrote the manuscript in consultation with AS and CM. All authors contributed to the article and approved the submitted version.

## Funding

This work was supported by a Starting Grant from the European Research Council (ERC) under the European Union’s Horizon 2020 research and innovation programme, G.A. No. 804388, wHiSPER.
